# The clinical application of transarterial embolization via radial artery in hemorrhagic diseases in obstetrics and gynecology

**DOI:** 10.3389/fmed.2023.1273179

**Published:** 2023-10-19

**Authors:** Xueqin Ma, Hao Chen, Xiaoping Luo, Jin Wang, Baolin Zhou, Xi Liu

**Affiliations:** Department of Radiology, Second Affiliated Hospital of Chongqing Medical University, Chongqing, China

**Keywords:** transradial approach (TRA), transfemoral approach (TFA), uterine artery embolization (UAE), Transarterial embolization (TAE), quality of life

## Abstract

**Purpose:**

The present study aimed to explore the feasibility and safety of Transarterial embolization (TAE) in the treatment of obstetrics and gynecological hemorrhagic diseases transradial approach (TRA) compared to transfemoral approach (TFA).

**Methods:**

This cohort study was conducted on patients with obstetrics and gynecology bleeding diseases from June 2021 to November 2022. Clinical characteristics of the patients were comparable between the two groups. The success rate of puncture and embolization, radiation dose, operation time, fluoroscopy time (FT), as well as complications of each patient were recorded and then retrospectively analyzed the data. The European Five-dimensional Health Scale (ED-5Q) and visual analog scale (VAS) were used to assess the quality of life (QOL) on the day of discharge and 30 days after surgery between the two groups.

**Results:**

A total of 71 patients undergoing TAE were allocated to the TRA (*n* = 31) or TFA (*n* = 40) group in this study. Puncture and embolization were completed in all patients. Compared to the TFA group, the radiation dose of the TRA group (343.89 ± 108.81 mGy vs. 469.29 ± 198.66 mGy; *p* = 0.029) is significantly reduced. Minor complications occurred in only one patient (3.2%) in the TRA group. The surgery-related quality of life EQ-5D index score on the day of discharge in the TRA group (0.72 ± 0.12 vs. 0.65 ± 0.11; *p* = 0.017) was significantly higher than that in the TFA group, and the VAS score (2.55 ± 0.62 vs. 2.95 ± 0.85; *p* = 0.025) of catheter site discomfort was significantly lower in the TRA group were than in the TFA group, but no significant difference was observed in the QOL assessment at 30 days post-surgery.

**Conclusion:**

Transradial approach TAE has comparable efficacy and safety to TFA TAE in treating obstetrics and gynecological bleeding diseases. This access can improve patient QOL without affecting surgical safety.

## 1. Introduction

Hemorrhagic diseases in obstetrics and gynecology are common in clinics, with complex mechanisms and many predisposing factors, and postpartum hemorrhage is a common cause. The main clinical manifestation of the patient is acute bleeding, which will seriously threaten the life, health and safety of the patient if not treated in time. Postpartum hemorrhage is the main cause of maternal death and serious complications worldwide, and about a quarter of maternal deaths are caused by postpartum hemorrhage (1, 2).

Transcatheter artery embolization (TAE) especially uterine artery embolization (UAE) is widely used in the treatment of gynecological and gynecological diseases, such as uterine fibroids, postpartum hemorrhage, scar pregnancy, and pelvic trauma, due to its advantages of small trauma, rapid hemostasis, and accurate positioning (3). Some studies have pointed out that the most common responsible vessels in Pelvic artery embolization (PAE) of postpartum hemorrhage are uterine artery, followed by internal iliac artery, circular ligament artery, and ovarian artery (4). Transfemoral approach (TFA) is the most classic and commonly used route in TAE. Femoral artery has strong vascular pulse, easy to touch, relatively fixed and thick diameter, high success rate of puncture, and easy to operate by interventional radiologists, but it requires lower limb immobilization and long bed rest after the operation, causing inconvenience to patients’ life and nursing (5).

Transradial approach (TRA) has been widely recognized in the field of cardiac intervention due to its advantages of reducing local complications, improving patient comfort, and reducing hospitalization costs (6–8). The success of TRA in coronary intervention has prompted a shift toward non-coronary research (9–11). Important factors that hinder the selection of TRA by interventional radiologists include: concerns about long learning curve, lack of training, long surgical time, potential cerebrovascular complications, and subjective perception of long exposure time (12, 13). Several recent studies have shown that in terms of procedure variables, clinical outcomes, and complication rate, transradial UAE is almost equivalent to transfemoral UAE (14–16). So far, however, most of the studies have reported that those treated with UAE are uterine fibroid patients. Therefore, the present study aimed to explore the feasibility and safety of TRA and TFA in the treatment of gynecological and obstetrical hemorrhagic diseases in TAE.

## 2. Methods

### 2.1. Study design and patients

**Figure 1:**
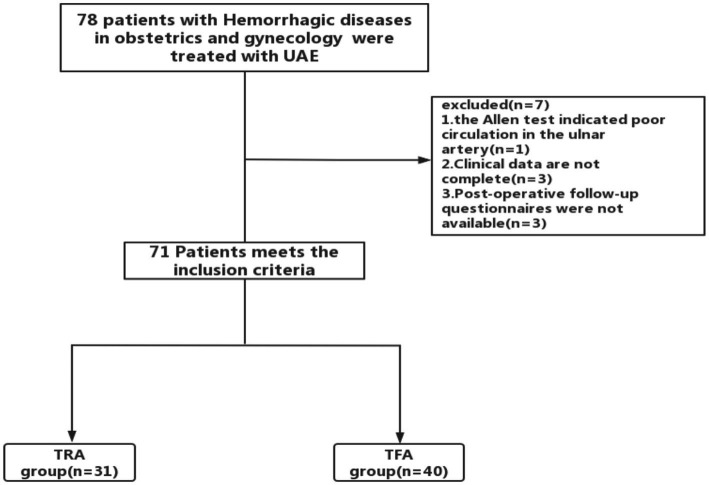
Flowchart of patient participation.

From June 2021 to November 2022, 78 patients who underwent TAE due to gynecological and obstetrical bleeding diseases were initially selected at our institution. The inclusion criteria were as follows: (1) Patients diagnosed with hemorrhagic diseases in obstetrics and gynecology by ultrasound and magnetic resonance imaging (MRI) and planned to undergo PAE. (2) Patients with hemorrhage after emergency obstetrics and gynecology surgery. (3) Age between 18 and 75 years. The exclusion criteria were as follows: (1) Preoperative assessment failed to complete the radial or femoral artery puncture, including the TRA: Allen test showed poor circulation of the ulnar and radial arteries; a history of severe vascular curvature or radial artery occlusion (radial artery occlusion, RAO); the need for dialysis; Raynaud’s phenomenon; TFA: postural restriction and bilateral iliofemoral bypass graft and bilateral iliofemoral or major iliofemoral occlusion. (2) Unable to complete the questionnaire at a later stage, which makes evaluation and comparison challenging. (3) Patients with uncontrolled high blood pressure, severe hepatic impairment, congestive heart failure, renal failure, or ventricular arrhythmias. At our institution, both radial and femoral access are routinely provided to patients. The selection of the vascular access site was determined by a multidisciplinary team after conducting a comprehensive evaluation, taking into account the preferences expressed by the patient and the vascular access available. Written informed consent was obtained from each patient enrolled in the study (see Figure 1).

### 2.2. Intervention

All procedures were performed by two experienced interventional radiologists (> 12 years of interventional experience and > 200 cases of PAE).

#### 2.2.1. TRA procedure

**Figure 2:**
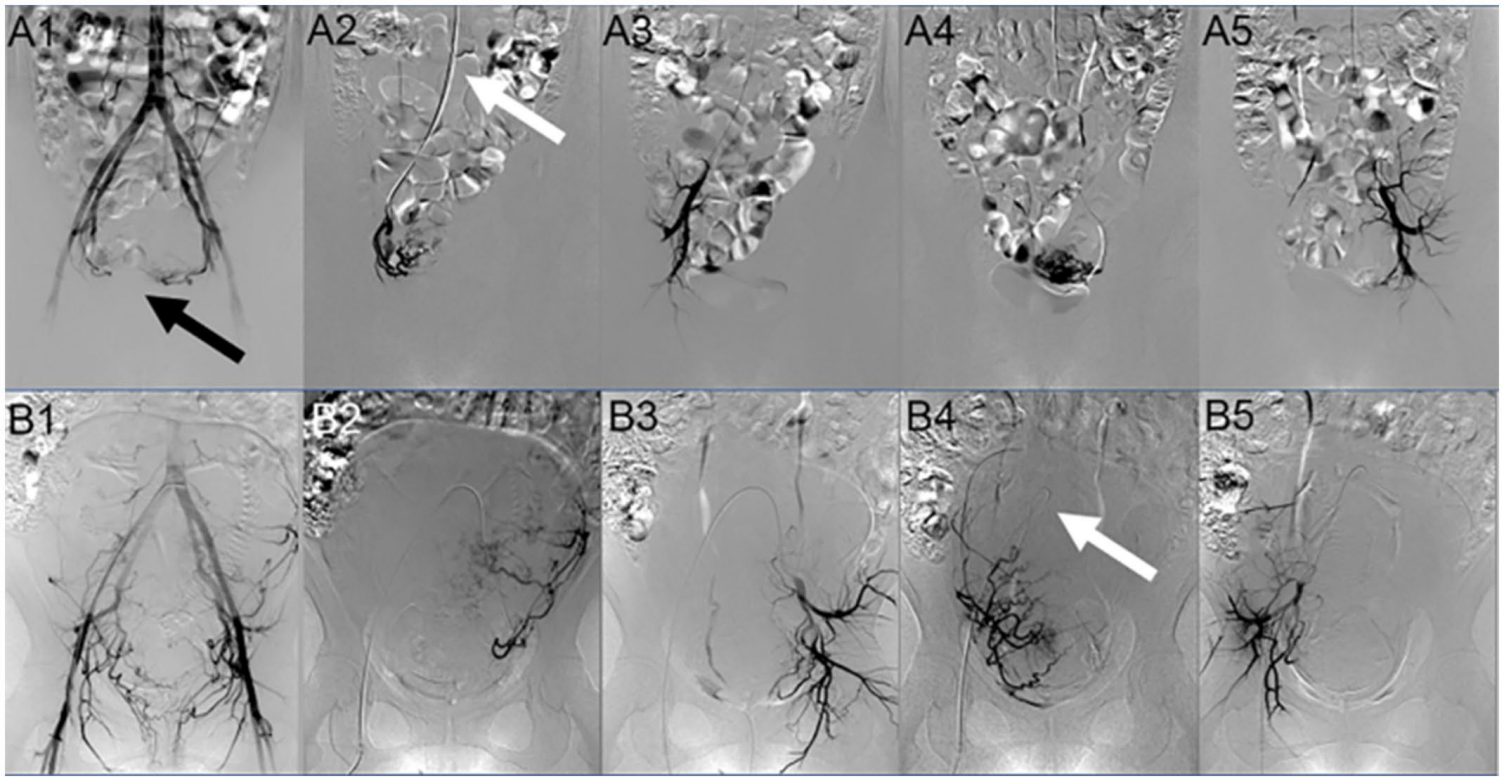
Digital subtraction angiography (DSA) image comparison of two paths. Image **(A)** is DSA image of the TRA group patient. **A**_**1**_ shows the angiographic image of the lower segment of the abdominal aorta. The bilateral uterine arteries are significantly thickened and tortuous, and the opening and shape of the uterine arteries are clear (as shown by the black arrow); **A**_**2**_ is the angiographic image of the right uterine artery after intubation; **A**_**3**_ is the angiographic image after embolization, showing that the tortuous and thickened uterine artery is not developed; **A**_**4**_ is the angiographic image of the left uterine artery after intubation; and **A**_**5**_ is the angiographic image after embolization. The tortuous and thickened uterine artery is not developed (as shown by the white arrow, the catheter is placed along the abdominal aorta-iliac artery to prevent it from being folded due to the large angle). Image **(B)** is the DSA image of the TFA group patient. **B**_**1**_ shows the angiographic image of the lower segment of the abdominal aorta. The bilateral uterine arteries are significantly thickened and tortuous, and the opening and shape of the uterine arteries are clear; **B**_**2**_ is the angiographic image of the left uterine artery from intubation; **B**_**3**_ is the angiographic image after embolization. The tortuous and thickened uterine artery is not developed; **B**_**4**_ is the angiography image of the right uterine artery after intubation [as shown by the white arrow, the catheter was looped (a loop was formed between the aorta and the right uterine art)]. At this time, it may be challenging to operate, and the catheter cannot be removed due to the large angle; and **B**_**5**_ is the angiographic image after embolization. The tortuous and thickened uterine artery is not developed.

In the TRA group, all patients underwent Allen’s test, and the return of blood circulation was observed and recorded. The patient was placed on the angiography table, and the right arm (*N* = 29) or left arm (*N* = 2) was placed on an arm board for support. An equivalent of 3–5 mL of 2% lidocaine was administered to the patient in the subcutaneous tissues around the radial artery approximately 2 cm proximal to the radial styloid over a distance of 3–4 cm. Then, a 21-G needle was used to puncture the anterior wall of the artery. After the blood ejected from the tail of the puncture needle, a 0.021-in guidewire was inserted into the artery, and a 4–6 Fr catheter sheath was placed appropriately. Then, 100 mg of nitroglycerin mixed with 2,000 IU heparin was given, and the vascular sheath was washed for 20 s to prevent vasospasm and thrombosis. MPA catheter (diameter 4 F, length 125 cm, Cordis Corporation, Miami Lakes, United States), BEEN catheter (diameter 4 F, length 100 cm, Terumo corporation, Tokyo, Japan), and VERTEBRAL catheter (diameter 5 F, length 100 cm, Terumo corporation, Tokyo, Japan) was used for uterine arterial catheterization. Under fluoroscopy, the catheter with guide wire was inserted into the lower segment of the abdominal aorta for angiography. Imaging showed that bilateral uterine arteries are significantly thickened and tortuous, local blood vessels are disordered, and some of them show signs of contrast medium overflow. After defining the shape and opening of the uterine artery, the 2.4 Fr or 2.8 Fr microcatheter (Merit Medical, South Jordan, UT) was inserted directly into the unilateral uterine artery with the cooperation of the micro guidewire (Merit Medical, South Jordan, UT); this phenomenon was confirmed by angiography. According to the experience of the operator and the type of disease, an appropriate amount of embolic agent was injected toward the stagnation of blood flow (the end point of embolization was defined as the slow blood flow of three cardiac cycles). Then, the catheter was withdrawn, and intubation was performed into the opposite uterine artery. After confirmation by angiography, an appropriate amount of embolic agent reverted the stagnation of blood flow. In case of pain or vasospasm during the operation, 1 mL lidocaine was injected. Finally, angiography was performed at the lower segment of the abdominal aorta to confirm the effect of embolization and prevent incomplete embolization (Figure 2A). Subsequently, the catheter and vessel sheath was removed successively, and a gauze and artery compression hemostat was used to stop bleeding. After confirming the normal pulsation of the distal palmar artery, the patient back was sent back to the ward safely.

#### 2.2.2. TFA procedure

In the TFA group, femoral artery puncture was performed by the Seldinger technique. After successful puncture, a 5 F catheter sheath (Prelude, Merit Medical) was utilized in all cases. Then, 100 mg of nitroglycerin mixed with 2,000 IU heparin was given, and the vascular sheath was washed for 20 s to prevent vasospasm and thrombosis. Uterine arterial catheterization was performed using Cobra catheter (5 F, length 80 cm, Cook Incorporated, Bloomington, United States) or VERTEBRAL catheter (diameter 5 F, length 100 cm, Terumo Corporation, Tokyo, Japan). Under fluoroscopy, the catheter was inserted into the lower segment of the abdominal aorta for posterior angiography (the imaging signs were basically the same as before). After the shape and opening of the uterine artery were confirmed, a 2.4 Fr or 2.7 Fr microcatheter (Terumo, Somerset, NJ, United States) was inserted directly into the left uterine artery with the help of a micro guidewire (Terumo, Somerset, NJ, United States). The technique used for embolization was the same as the radial method. After successful embolization of the left uterine artery, a loop was formed in the aorta, and the ipsilateral uterine artery was catheterized with the cooperation of the guide wire. This positioning was confirmed, following which the catheter was also embolized to the stagnation of blood flow. Finally, angiography was performed at the lower segment of the abdominal aorta to confirm the embolic effect (Figure 2B). Then, the catheter was pulled out and the sheath was removed, the bleeding was stopped and banded, the pulsation of dorsalis pedis artery was confirmed, and the patient was sent back to the ward.

In order to identify the site of bleeding and obtain selective embolization, selective angiography is mandatory. In the absence of a source of bleeding, uterine artery embolization was performed, followed by angiography in the lower abdominal aorta to identify and embolize other bleeding vessels. We named the embolization of only the uterine artery as vessel I, and the embolization of other vessels as vessel II. All the above operations were performed in the dedicated angiography suite. Digital subtraction angiography (DSA) is limited to the acquisition before and after UAE. The use of X-ray fluoroscopy is minimized.

### 2.3. Study endpoint

The primary endpoint of this study was the success rate of puncture and embolization and complications, while the secondary endpoints were procedural variables and quality of life (QOL). The insertion of a vascular sheath into the artery was regarded as a successful puncture, and the catheter entry into the target vessel and completion of the subsequent surgery was regarded as a successful operation. The procedure variables included radiation dose, total fluoroscopy time (FT), FT1 [puncture-lower abdominal aorta time (min)], FT2 [lower abdominal aorta-one uterine artery time (min)], FT3 [embolization completion-contralateral uterine artery time (min)], and operation time. The demographic information, medical and surgical history, and clinical symptoms were recorded prospectively in the medical electronic medical records of the patients. This information is obtained from the image archive and program reports.

Quality of life was assessed using the European Quality of Life-5 Dimensions (EQ-5D) questionnaire, and the EQ-5D health index was calculated based on the choices made by the study subjects for each dimension (17). A higher score on the health index indicates a better QOL. The patient’s surgery-related QOL was assessed on the day of discharge and on the 30^th^ postoperatively using the EQ-5D questionnaire (for use in China), and the patient was asked about discomfort at the site of catheterization using the visual analog score (VAS) scale (0 for no pain, 10 for the most severe pain that was unbearable).

Radial artery catheterization is an invasive procedure that can lead to many complications. The radial artery was examined on the day after the operation to evaluate the access site. In the follow-up, we assessed whether the patient has any common postoperative complications, including major (major hematoma, pseudoaneurysm, limb ischemia, arteriovenous fistula, nerve damage, hand dysfunction, and any access site complication requiring open surgical intervention) and minor (minor hematoma and radial artery occlusion without evidence of distal ischemia) events (18).

### 2.4. Statistical analysis

SPSS commercial software was used for all data analysis (SPSS statistics version 25.0, IBM, Chicago, IL, United States). The comparisons between the two groups were performed using the *t*-test for measurement data, expressed as mean ± standard deviation, while the chi-square test was used for categorical variable data (expressed in percentage). Values of *p* < 0.05 were defined as statistically significant.

## 3. Results

### 3.1. Patient characteristics

During the study period, 78 patients were screened for enrollment in the study. Subsequently, seven patients were excluded: one patient had poor radial collateral circulation, three patients had incomplete clinical data, and postoperative follow-up questionnaires were not available for three patients. Finally, 71 patients were enrolled in the study: 31 patients were included in the TRA group and 40 were included in the TFA group (Figure 1). In this study, after bilateral uterine artery embolization, one case in TRA group formed a communicating branch between the right uterine artery and the bladder artery, and the other case embolized the superior vesical artery and the left external iliac artery. In the TFA group, four patients had embolization of other vessels besides the uterine artery, including vesical artery, internal pudendal artery, obturator artery, superior rectal artery, and internal pudendal artery. The demographics and clinical characteristics of the patients were similar between the two groups (*p* > 0 0.05; Table 1).

**Table 1:** Demographic characteristics.

	TRA (*n* = 31)	TFA (*n* = 40)	*p*
Age (years)	35.52 ± 8.93	34.13 ± 4.96	0.440
Height (cm)	157.03 ± 3.50	156.73 ± 2.65	0.686
Weight (kg)	54.77 ± 5.21	56.00 ± 2.97	0.248
BMI (kg/m^2^)	22.20 ± 1.86	22.79 ± 0.84	0.108
Etiology			
Ectopic pregnancy	28 (90.3)	35 (87.5)	0.709
Genital tract malignancies	3 (9.7)	3 (7.5)	0.744
Fibroids with vaginal bleeding	0 (0)	2 (5.0)	0.207
Arterio-venous mal-formations	0 (0)	0 (0)	0.999
Embolized vessel type			0.594
Vessel I	29 (93.5)	36 (90.0)	
Vessel II	2 (6.5)	4 (10.0)	
Platelet count (No. × 10^3^/μL)	221.45 ± 74.07	189.33 ± 60.37	0.150
Hemoglobin (g/dL)	108.52 ± 17.99	106.13 ± 18.48	0.585
Erythrocyte (No. ×10^9^/μL)	3.69 ± 0.68	3.61 ± 0.69	0.612
Type of operation			0.946
Emergency operation	25 (80.6)	33 (82.5)	
Elective operation	6 (19.4)	7 (17.5)	

Values are *n* (%), mean ± standard deviation or median (interquartile range); BMI, Body mass index; TFA, Transfemoral approach; and TRA, Transradial approach.

### 3.2. Safety and complications

Embolization and puncture were completed successfully in all patients. Therefore, the success rate of puncture and operation was 100%. No rebleeding or major vascular complications occurred in both groups during the postoperative follow-up. Only one case (3.2%; *p* = 0.253) of radial artery thrombosis occurred in the TRA group, while no major pathway site complications occurred in the TFA group (Table 2). Although no statistically significant difference was observed in the following evaluation indicators (mobility, self-care, manual activities, pain/discomfort, and anxiety/expression) between the two groups at the time of discharge, the score of EQ-5D index related to surgery in the TRA group (0.72 ± 0.12) was significantly higher than that in TFA group (0.65 ± 0.11; *p* = 0.017; Table 3). In addition, the VAS score of discomfort at the catheter site in the TRA group was significantly lower than that in the TFA group (2.55 ± 0.62 vs. 2.95 ± 0.85; *p* = 0.024), indicating a significant improvement in the QOL of patients in the early stage after the operation (Table 4). On the other hand, 30 days after the operation, the difference between the two groups (EQ-5D index: 0.85 ± 0.13 vs. 0.82 ± 0.14; *p* = 0.467) and VAS (1.48 ± 0.51 vs. 1.70 ± 0.56; *p* = 0.095) did not differ significantly.

**Table 2:** Safety outcomes.

Vascular access-site complications	TRA (*n* = 31)	TFA (*n* = 40)
Major hematoma	0 (0)	0 (0)
Pseudoaneurysm	0 (0)	0 (0)
Limb ischemia	0 (0)	0 (0)
Arteriovenous fistula	0 (0)	0 (0)
Nerve damage	0 (0)	0 (0)
Radial artery occlusion	0 (0)	0 (0)
Radial artery thrombosis	1 (3.2)	0 (0)

Data presented as *n* (%). TFA, Transfemoral approach; TRA, Transradial approach.

**Table 3:** Discharge and 30-day EQ-5D scores according to access site.

Variable		Discharge	30-day				
		TRA (*n* = 31)	TFA (*n* = 40)	*p* value	TRA (*n* = 31)	TFA (*n* = 40)	*p* value
Mobility	No problems with walking	22 (71.0)	30 (75.0)	0.703	29 (93.5)	36 (90.0)	0.594
	Some problems with walking	9 (29.0)	10 (25.0)		2 (6.5)	4 (10.0)	
	Confined to bed	0 (0)	0 (0)		0 (0)	0 (0)	
Self-care	No problems with self-care	20 (64.5)	25 (62.5)	0.861	29 (93.5)	39 (97.5)	0.412
	Some problems with washing or dressing myself	11 (35.5)	15 (37.5)		2 (6.5)	1 (2.5)	
	Unable to self-wash or -dress	0 (0)	0 (0)		0 (0)	0 (0)	
Usual activities	No problems performing usual activities	15 (48.4)	19 (47.5)	0.941	28 (90.3)	39 (97.5)	0.193
	Some problems performing usual activities	16 (51.6)	21 (52.5)		3 (9.7)	1 (2.5)	
	Unable to perform usual activities	0 (0)	0 (0)		0 (0)	0 (0)	
Pain/discomfort	No pain/discomfort	17 (54.8)	15 (37.5)	0.145	21 (67.7)	23 (57.5)	0.378
	Moderate pain/discomfort	14 (45.2)	25 (62.5)		10 (32.3)	17 (42.5)	
	Extreme pain/discomfort	0 (0)	0 (0)		0 (0)	0 (0)	
Anxiety/depression	Not anxious or depressed	10 (32.3)	8 (20.0)	0.126	20 (64.5)	18 (45.0)	0.102
	Moderately anxious or depressed	21 (67.7)	28 (70.0)		11 (35.5)	22 (55.0)	
	Extremely anxious or depressed	0 (0)	4 (10.0)		0 (0)	0 (0)	

Data presented as *n* (%). EQ-5D, European Quality of Life-5 Dimensions; TFA, Transfemoral approach; and TRA, Transradial approach.

**Table 4:** QoL summary measures.

Measure	TRA (*n* = 31)	TFA (*n* = 40)	*p* value
EQ-5D index score			
Hospital discharge	0.72 ± 0.12	0.65 ± 0.11	0.017
30-day	0.85 ± 0.13	0.82 ± 0.14	0.467
Vas score			
Hospital discharge	2.55 ± 0.62	2.95 ± 0.85	0.024
30-day	1.48 ± 0.51	1.70 ± 0.56	0.095

Values are mean ± standard deviation or median (interquartile range). VAS, Visual analog scale; TFA, Transfemoral approach; and TRA, Transradial approach.

### 3.3. Program variables

The radiation dose received during the operation was significantly lower in the TRA group (343.89 ± 108.81 mGy) than in the TFA group (469.29 ± 198.66 mGy; *p* = 0.029). Concurrently, FT2 (4.75 ± 1.71 min) and FT3 (4.09 ± 1.84 min) were lower in the TRA group than in the TFA group (5.94 ± 2.27 min; *p* = 0.014) and (5.60 ± 1.83 min; *p* = 0.001; Table 5). The FT1 in the TRA group (6.04 ± 2.04 min) was longer than that of the TFA group (4.02 ± 1.17 min; *p* < 0.001). In addition, the operation time of the TRA group (51.02 ± 8.34 min) was not significantly different from that of the TFA group (47.57 ± 9.77 min; *p* = 0.114). The total fluoroscopy time of the TRA group (19.69 ± 9.85 min) and TFA group (16.66 ± 7.84 min) did not differ markedly (*p* = 0.165). During UAE, 30 (96.8%) patients in the TRA group and 31 (85%) patients in the TFA group used microcatheters for super-selective catheterization and embolization (*p* = 0.099). In the TRA group, the following catheters were used for uterine arterial catheterization: MPA catheter in seven patients (22.6%), BEEN catheter in 14 patients (45.2%) and VERTEBRAL catheter in 10 patients (32.2%). In the TFA group, the following catheters were used for uterine arterial catheterization: 35 patients (82.5%) used The VERTEBRAL catheter and five patients (17.5%) used the Cobra catheter.

**Table 5:** Procedural variables.

	TRA (*n* = 31)	TFA (*n* = 40)	*p*
X-ray radiation	343.89 ± 108.81	469.29 ± 198.66	0.029
Mean total FT (min)	19.69 ± 9.85	16.66 ± 7.84	0.165
FT1 (min)	6.04 ± 2.04	4.02 ± 1.17	<0.001
FT2 (min)	4.75 ± 1.71	5.94 ± 2.27	0.014
FT3 (min)	4.09 ± 1.84	5.60 ± 1.83	0.001
Microcatheter used	30	34	0.099
Operation time	51.02 ± 8.34	47.57 ± 9.77	0.114
Technical success	31	40	1
Sheath diameter, 4F/5F/6F	10 (32.3%)/21 (67.7%)/0 (0%)	0 (0%)/40 (100%)/0 (0%)	

FT1 = FT from the beginning of access artery puncture to the lower segment of the abdominal aorta. FT2 = FT to embolization of first uterine artery with stasis as the endpoint. FT3 = FT from the end of the first UAE to catheterization of the contralateral uterine artery. SD, Standard deviation; FT, Fluoroscopic time.

## 4. Discussion

The present study of 71 patients with gynecological and obstetrical bleeding diseases showed that the feasibility, safety, operation time, and fluoroscopy time of TRA TAE were similar to those of traditional TFA, which can effectively stop bleeding without causing severe postoperative complications, simultaneously improving the QOL of patients in the early postoperative period and provide better medical experience to patients.

Even if the concept of embolization is the same, different sites of entry may have unexpected effects on patient outcomes and experience. TRA provides a safe, effective and patient-centered new model, which represents best practice in the changing healthcare environment (11). In a systematic review of UAE of TRA (18), the technical success rate of TRA was shown to be 97–100%, which is in line with the published SIR Quality improvement benchmark of 95% (19), and 100% technical success rate was reported in this study, which is also consistent with the research results. Regarding access site complications, Khayrutdinov et al. (16) reported a secondary complication rate of 11.53% in the TRA group and 17.3% in the TFA group. The rate of major (0 vs. 2.7% *p* = 0.37) complications was comparable between the two groups. In this study, minor pathway-related complications were not significantly different between the TRA and TFA groups.

Different from the results of local intubation time of Mortensen et al. (20), the uterine artery catheterization time FT2 and FT3 in the TRA group were significantly reduced, which indicated the advantage of the radial artery pathway in the time saving of intubation in the above two stages. In addition, these two time periods are the most critical and time-consuming in the process of embolization and hemostasis. In some cases, non-uterine arteries may be the main source of postpartum hemorrhage, and their detection and selective embolization are important for successful hemostasis. Most of them are attached at an acute Angle to the abdominal aorta or partially form collateral or co-trunk with the main uterine artery, so the radial artery route is easier to intubate in the lower segment. As described in the previous surgical procedure, the TFA needs the catheter to form a loop in the aorta, for those who are not rich in experience, it will take a certain amount of time. If the blood vessel is too twisted, resulting in loop formation and difficult intubation, it will greatly extend our time. On the other hand, the FT1 time in TRA group is longer than that in TFA group, which may be caused by the following factors, including: the diameter of radial artery is smaller than that of femoral artery, which is difficult to puncture; After successful puncture, the intubation route to the lower segment of the abdominal aorta is longer (the length of the catheter in TRA group is 120 cm > the length of the catheter in TFA group is 65 cm), and there is interference from the larger branches of the upper limb-shoulder-neck artery and aortic arch in the route (such as axial artery, vertical artery, and arteria carotis communication) (21). These factors above increase the complexity of the operator, not only because the extra distance must be covered, but also because the pushability and twist of the catheter are reduced (9).

At the same time, the experience of operators is also an important influencing factor. According to a study on the experience of operators who underwent transcatheter arterial embolization of liver cancer, the threshold for overcoming the learning curve is 20 cases (22). Although the TRA path process is relatively time-consuming, there is no significant difference between the two groups in the results of the two indicators of operation time (*p* = 0.101) and fluoroscopy time (*p* = 0.184), which also indirectly verifies the shorter results of FT2 and FT3 in two time periods that are important for rapid hemostasis. After all, the TFA loop is also complex and time-consuming, and there is a risk of catheter discount.

It is worth mentioning that the radiation dose of the TRA group (343.89 ± 108.81 vs. 469.29 ± 198.66 mGy; *p* = 0.029) in this study results was significantly reduced, which is consistent with the results of Khayrutdinov et al. (16) We did not observe that a longer fluoroscopy time would translate into a higher radiation dose. The reasonable explanation is that some emergency patients have a large amount of bleeding, which makes it impossible to ensure that we can always operate on the same DSA machine. The difference in the radiation dose of the rays sent by each machine and the different degree of vascular opening and distortion make the intraoperative imaging times different. The previous study on transcatheter arterial chemoembolization (TACE) of TRA (23) showed that the radiation dose of the operator was the smallest when the left radial artery and the left arm were extended about 70–90°. Our study did not directly measure the radiation exposure of the operator, but this may be a direction of future research.

Previous comparative study and analysis showed that coronary artery catheterization using the left radial artery pathway can reduce the risk of cerebral embolism (24), but it is not clear whether it can be extrapolated to the UAE procedure, because patients with heart disease often have a high degree of atherosclerosis, and more aortic arch branches need to be operated. Although there is a risk of thrombus shedding through the radial artery, the risk is very small under the condition of short time operation and reasonable heparinization (19). None of the patients in this study showed neurological symptoms or functional defects during the follow-up period, although it is likely that the sample size of the study is too small or subclinical emboli were not found. A miscarriage patient in the TRA group developed radial artery thrombosis after operation and improved after anticoagulation treatment. This is the first case of UAE through the radial artery by the operator. The reasonable explanation is related to the learning curve and the small diameter of the radial artery.

For postoperative pain management, the transradial approach is more helpful for patients with transcatheter artery embolism, because their legs can move freely during and after surgery, so the pain in the lower abdomen can be relieved by sitting up in a comfortable position or curling up their legs. Although the choice of entry site has no direct impact on post-embolism syndrome-related pain, for our patients, women do not need to expose private sites during the operation, do not need to lie still after the operation, immediately resume various normal physiological activities and follow-up treatment, avoid the difficulty of defecation caused by fixed body position, remove the radial artery compression device within 2 h after the operation, and significantly improve their medical experience. TRA can improve patients’ overall satisfaction, and 73–79% of patients are more inclined to TRA when receiving interventional therapy (11, 25, 26). In our study, the EQ-5D index score and the VAS score of the intubation site discomfort of patients in the TRA group were better than those in the TFA group (*p* = 0.017, *p* = 0.024) at the time of discharge, suggesting that the quality of life of patients after surgery was higher, which was consistent with the previous research results of Basile (27) and others, and the VAS score of the intubation site discomfort of patients in the TRA group was better than that in the TFA group (*p* < 0.001) 30 days after discharge, which showed the advantage of early recovery in the TRA group.

However, our study has several limitations. First, the single-center study had a small sample size. Second, this study did not focus on assessing other potential benefits of TRA technology, such as length of hospital stay and cost-effectiveness evaluation. We also did not routinely record operator radiation doses. Third, the most common complication of TRA is RAO formation (28), which is asymptomatic in most cases, and the lack of Doppler ultrasound to assess subclinical arterial thrombosis in this study may lead to underestimation of complications.

## 5. Conclusion

Transradial approach is a safe and effective approach in obstetrical and gynecological patients undergoing TAE treatment for hemorrhagic diseases, as it improves the quality of life of patients in the early postoperative period without affecting surgical safety, which is worth promoting. Larger and longer prospective randomized trials are needed to validate the potential clinical benefits of this novel transradial approach in TAE.

## Data availability statement

The data presented in this study are available on request from the corresponding author. The data are not publicly available due to privacy issues.

## Ethics statement

The studies involving humans were approved by the Ethics Committee of the Second Affiliated Hospital of Chongqing Medical University (Date 2023-06-15/No. 95/2023). The studies were conducted in accordance with the local legislation and institutional requirements. The participants provided their written informed consent to participate in this study. Consent for publication was obtained for every individual person’s data included.

## Author contributions

XM: Data curation, Methodology, Writing – review & editing, Conceptualization, Formal analysis, Investigation, Project administration, Software, Writing – original draft. HC: Conceptualization, Data curation, Investigation, Writing – original draft, Writing – review & editing, Supervision, Validation. XLu: Data curation, Investigation, Supervision, Validation, Writing – review & editing, Formal analysis, Project administration. JW: Data curation, Investigation, Supervision, Validation, Writing – review & editing, Resources, Software. BZ: Data curation, Supervision, Writing – review & editing, Methodology. XLi: Data curation, Methodology, Supervision, Writing – review & editing, Validation.
